# Birth after Death of Mother

**Published:** 1890-06

**Authors:** 


BIRTH AFTER DEATH OF MOTHER.—Dr. I. A. Shute, of Opelousas, La., in the August number of the *World*, gives us an account of birth after death, and asks, "Is there anything similar on record?" Yes, I would say in reply, numerous cases are on record of births taken place shortly after the mother has died and been prepared for burial. The uterus retains its power of contraction even after the event of death. Cazeaux, in his work of Midwifery, speaks of it Here are his words, page 151, 5th edition:

"The contractibility of the uterus, like that of all the viscera of organic life, is retained for some time after death, and thus serves to explain the occasional expulsion of a fetus several hours subsequent to the decease of the mother."

So, we can see that, although it is comparatively a rare event, it is still possible as long as the contractibility remains.—*Medical World.*

